# Group-Based Trajectory Analysis for Postpartum Depression Symptoms among Chinese Primiparous Women

**DOI:** 10.3390/jcm11216249

**Published:** 2022-10-23

**Authors:** Juan Xiong, Qiyu Fang, Lingling Huang, Xinyi Yan, Xujuan Zheng

**Affiliations:** 1 Health Science Centre, Shenzhen University, Shenzhen 518060, China; 2 School of Nursing, LKS Faculty of Medicine, The University of Hongkong, Hong Kong, China

**Keywords:** postpartum depression, trajectory analysis, primiparous women, group-based trajectory model

## Abstract

Background: Subgroups of individuals sharing similar patterns of postpartum depression (PPD) among Chinese women are unknown thus far. Using a group-based trajectory model, this study aimed to explore the subgroups of Chinese primiparous women that share similar patterns of PPD and to explore the predictors of PPD trajectory membership over the course of the first six months postpartum. Methods: In total, 674 first-time Chinese mothers were recruited, and their depression status was assessed using the Edinburgh Postnatal Depression Scale (EPDS) at four time points. Findings: Around 18.0% of participants belonging to Group 1 labeled as “few or no symptoms” remained stable, with an EPDS score of less than 5 during a six-month postpartum period. Almost one-third of subjects fell within the second trajectory, labeled “subclinical but present symptoms”, and peaked into the range of mild PPD but mostly stayed in the minimal range and had few or no PPD symptoms. Group 3 included 31.2% of women labeled “minor PPD status”, and their mean EPDS scores increased to a peak of 14.66 at six weeks postpartum. Group 4, with “major PPD status”, comprised 19.2% of the population, and the mean EPDS scores dramatically increased, reaching a peak of 19.59 at 12 weeks postpartum. Fewer types of support and not attending parenting training were associated with membership in the minor and major PPD status trajectories. Conclusions: Almost half of the Chinese new mothers in the study were found to fall into the two groups with minor or major PPD status trajectories, who should be given more attention and awareness from health professionals and researchers. Understanding predictors of group membership could help health providers to identify folks to prioritize getting connected to care as well as forming targeted interventions. Less degree of received support and not attending parenting training were identified to predict PPD trajectory membership. The regular, routine screening of PPD should be conducted at least 12 weeks postpartum, especially for new mothers in the major PPD status trajectory.

## 1. Introduction

Postpartum depression (PPD), also known as postnatal depression (PND), refers to any case of major or sub-clinical depression that occurs in the postpartum period [1]. In addition to common depressive symptoms, i.e., low mood, lack of energy, pessimism, sleeplessness, inattention, low self-esteem, irritability, and suicidal thoughts, women with PPD feel ill-equipped to care for their newborns [2,3,4,5]. For the last decade, PPD has been regarded as a significant public health problem worldwide because of the high prevalence and indiscriminate harm to new mothers, their infants, and family members [4,6].

Varied research has found that the worldwide incidence of PPD ranges from 10% to greater than 25% within the first year after childbirth [7,8,9]. For instance, in high-income countries, approximately one-fifth of new mothers experience PPD symptoms. By contrast, PPD affects more than one-quarter of women in developing countries [10]. A recent systematic review evaluated 122 studies and found that PPD negatively influenced the physical and mental well-being of new mothers, the health and development of infants, and relationships with family members [11]. For example, PPD has been a leading cause of maternal morbidity and mortality in different countries [4]. Furthermore, PPD seriously impairs mother–infant attachment [12] and maternal parenting confidence and capability [13,14], which could interfere with the infant’s emotional and behavioral development in the long term [12,15]. Moreover, PPD has detrimental consequences for the marital relationship and the psychological well-being of close family members [15,16].

There have been extensive longitudinal studies to explore the incidence of PPD and its risk factors at different time points, such as six weeks postpartum, 12 weeks postpartum, and 24 weeks postpartum. Longitudinal data, referring to data with a time-based dimension, provide the empirical foundation for the analysis of developmental trajectories [17]. Trajectory is used to describe the progression of any phenomenon. Once different trajectories are identified, predictors of trajectory membership can be determined. It was estimated that understanding predictors of group membership could help health providers to identify folks to prioritize getting them connected to care. Furthermore, such predictors may be useful as targets for interventions tailored to patients belonging to the clinically worst trajectories, which have profound clinical and practical significance [18]. Moreover, a group-based trajectory can highlight time points where intervention might be the most effective for different groups. However, subgroups of individuals that share similar patterns of postpartum depression symptoms among Chinese women on the mainland are unknown in the existing literature [19]. Fortunately, trajectory modeling approaches have been developed to address this challenge [18]. Via these approaches, individuals can be assigned to homogeneous subgroups (distinct trajectories) that are interpreted as representing similarities in given outcomes [18,20].

As the more practical choice of trajectory analysis, the group-based trajectory model (GBTM) offers a unique statistical method to categorize individuals into relatively homogeneous groups based on their trajectories [21,22]. The fundamental assumption of GBTM is that this analysis can identify subgroups that share similar trajectories, which distinguishes it from other longitudinal data analysis methods [22,23], such as mixed modeling and generalized estimating equations, which were designed to account for individual variability regarding a mean population trend. The GBTM assumption makes it well suited for healthcare research, owing to the well-recognized between-individual variation and between-group similarity in the development and progression of various human characteristics, disorders, and related symptoms [23]. Furthermore, it is important to study primiparous women as they are more likely to suffer from PPD than multiparas [13,14]. Therefore, based on the group-based trajectory model (GBTM), this research was conducted to identify the subgroups of Chinese primiparous women that share similar patterns of PPD and to explore the predictors of PPD trajectory membership to fill the research gap.

## 2. Materials and Methods

### 2.1. Study Design and Participants

The investigators were interested in understanding trajectories of symptoms in individuals without prior incidents of mental health conditions. A quantitative longitudinal study was conducted to explore the developmental PPD trajectories and the predictors of PPD trajectory membership among first-time Chinese mothers via GBTM. Upon approval by the Ethics Committee of the Medical School in Shenzhen University, in total, 674 primiparous women were recruited in their third trimester of pregnancy from the maternity wards of the three affiliated hospitals of xxx University in 2020–2021. The inclusion criteria of participants were as follows: first-time mothers having healthy babies; aged 18 years old or above; without depression and anxiety history; being able to complete the questionnaire. The exclusion criteria excluded women or their infants with serious physical or any other psychiatric disorder. Prior to participating in the study, all subjects provided electronic or written informed consent and were informed of the research aims and processes, and they had the freedom to withdraw from the research whenever they wished.

### 2.2. Instruments

The social–demographic and clinical data were collected with baseline questionnaires, including maternal age at childbirth, marital status, educational level, occupation, family income, delivery mode, whether the individual attended parent training at the hospital, infant gender, infant health, and infant fussiness, collected via self-report among the women. Parenting training in the current research refers to prenatal education conducted by well-trained health professionals in the hospital and lasting 15 to 30 min. The contents of parenting training courses comprised an introduction to childbirth, nutrition and exercise during pregnancy, mental health care, neonatal care, breastfeeding, postnatal care, etc.

Postpartum depression symptoms were assessed in the study using the Edinburgh Postnatal Depression Scale (EPDS), a reliable (Cronbach’s alpha coefficient = 0.87), validated (concurrent validity with Beck Depression Inventory = 0.79), ten-item instrument designed to screen for PPD [24]. The scale has been translated into more than 60 different languages and is widely used around the world [25]. The EPDS is scored from 0 to 30, and a lower score means better mental health status. In the current data set, the internal consistency of EPDS was 0.88. EPDS scores of 10 and 13 were the threshold scores for the symptoms of minor postnatal depression and major postnatal depression, respectively [26,27,28].

The various types of social support that women received after childbirth were measured by the Postpartum Social Support Scale (PSSS) in the Chinese version [29]. Women self-rated four types of support according to the 20-item scale, i.e., emotional support, material support, informational support, and evaluation of support, assigning a score of 0–3 depending on the response options of “never”, “rarely”, “sometimes”, and “often”. Each type of social support included five items. The higher score the women obtained, the more support the women received in the postpartum period. The internal consistency of this tool was 0.89 [29]. In the present research, Cronbach’s alpha coefficient of PSSS was 0.90.

### 2.3. Data Collection

The baseline questionnaires and EPDS were distributed to participants and collected by researchers face-to-face in the maternity wards on the third day postpartum, and these new mothers’ contact details were likewise collected. At six weeks postpartum, the electronic versions of the EPDS and PSSS were sent to participants via Wechat or email. At 12 weeks postpartum and 24 weeks postpartum, the electronic EPDS was likewise distributed to women. In order to decrease the attrition rate, Wechat or call reminders were given to participants a week before, a day before, a day after, and a week after each time point.

### 2.4. Statistical Analysis

Descriptive analysis was used to describe the social–demographic and clinical data. For continuous variables, mean and standard deviation (SD) were used, and for categorical variables, frequencies and percentages were used. The GBTM approach was conducted via SAS (version 9.4, Cary, NC, USA) software through the Proc Traj procedure [30,31] because it was found to be superior for identifying underlying longitudinal trajectories [32]. Proc Traj uses the maximum likelihood method to estimate parameters, including group sizes and shapes of trajectories. Trajectory memberships were then used as categorical variables in a multinomial logistic regression model to identify predictors of trajectory membership; outcomes are presented as odds ratios (ORs) and 95% confidence intervals (CI). A *p*-value < 0.05 meant that a difference was statistically significant.

Missing data are often a problem in longitudinal studies [33]. The distribution of missing data is shown in Table 1. Subjects with at least three data points (*n* = 435) were included in the GBTM analysis. Among these 435 patients, 131 subjects missed the last visit. The test developed by Jamshidian and Jalal [34] suggested our data to be missing completely at random (*p* = 0.393). For comparative purposes and to examine the effects of missing data on EPDS trajectory formation, participants with four visits (*n* = 304) likewise were used to identify the trajectory groups, and similar results were obtained with the findings among participants with three visits (*n* = 435). This indicated that there would be no difference between participants who fully responded and those who missed one visit.

### 2.5. Building the GBTM

**Figure 1:**
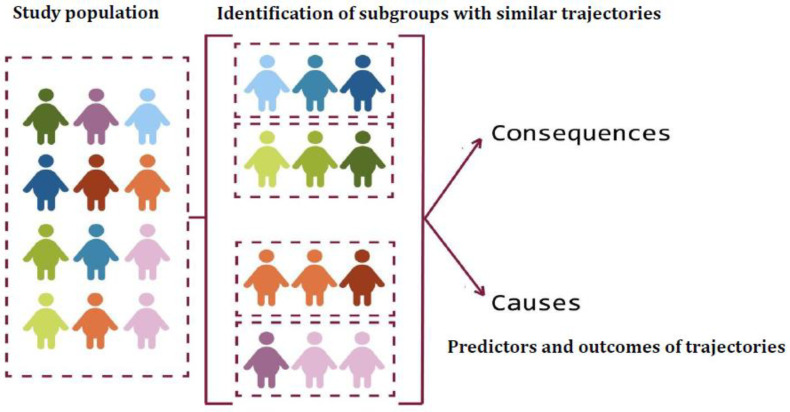
The fundamental assumptions of GBTM.

The GBTM assumes that the population is constituted of various subgroups with similar patterns of clinical symptoms, behaviors, or healthcare utilization [18,23]. The fundamental assumptions of the GBTM are illustrated in Figure 1. Once subgroups are identified, trajectory membership can be used as a dependent variable to identify predictors of health trajectories or an independent variable to explore their impact on future health outcomes [18].

Building the appropriate model was the key point in the GBTM analysis, which included the following two tasks. One task was to decide the optimal numbers of trajectory groups that best fit the data; the other was to determine the appropriate polynomial order that best characterized the shape of the trajectory. There are various criteria to assist in deciding on the best model, and the most commonly used is the Bayesian information criterion (BIC), which offers a good balance of goodness of fit to the data and model complexity [35]. Suppose comparing the two possible models with different trajectory numbers and/or polynomial order (trajectory shape), the model with the highest value of BIC should be chosen. However, when the number of trajectory groups or the shape of trajectories is changed, the BIC likewise would be changed, and the Bayes factor is the parameter to assess a meaningful change in BIC [35]. For two models, A and B, the Bayes factor is the ratio of the probability of model A being the correct model to the probability of model B being the correct model. When comparing two models, a 10-fold difference in the Bayes factor is considered a meaningful difference [35].

The process typically starts with a model consisting of one group that has the highest polynomial order; then, the group numbers are increased until the number of groups best fits the data, which is identified using a combination of the BIC and Bayes factors. Once the number of groups is identified, we then reduce the polynomial order until the highest-order polynomial for each group is significant at the confidence level alpha (α) = 0.05. In this research, the highest polynomial order was a cubic polynomial or third order because of the four data points measured in the research.

### 2.6. Evaluating Trajectory Model Fit

The evaluation of model adequacy is a significant step in GBTM analysis. The most useful diagnostic statistic is the average posterior probability (AvePP) of assignment for each group. If individuals are assigned to distinct groups with no ambiguity, the AvePP would be 1 for each group. Therefore, the closer the AvePP is to 1, the more adequate the model is in describing the data. It was recommended that an AvePP should be greater than 0.70 for all groups [23,35,36,37]. Another index of calculating trajectory model fit is the odds of correct classification (OCC) for each group, which is calculated as the ratio of the odds of correct classification based on the maximum probability classification rule (AvePP/1-AvePP) and the odds of correct classification based on random assignment, calculated using the proportion of individuals assigned to each group *π* (π/1 − π). Thus, if the maximum probability rule is not better than random guessing, the OCC would equal 1 for a given trajectory group. For a model that fits the data well, the OCC value is much greater than 1. Generally, an OCC of 5 or more is recommended for all groups. The other index is the value of |*π_j_* − *P_j_*|. *π_j_* denotes the population size of trajectory group *j* estimated by the model, and *P_j_* is the actual proportion of individuals that are assigned to group *j*. When these two quantities of *π_j_* and *P_j_* are similar, it indicates that the model fits the data well [33].

## 3. Results

### 3.1. Participant Profile

This study included 435 primiparous women with at least three visits, and the average age of this sample when giving birth was 25.56 (3.34) years. The social–demographic and clinical characteristics of the participants are summarized in Table 2.

### 3.2. Trajectory Model Development

As described above, the researchers began with a model that had one trajectory group and a cubic polynomial order (the highest in the research) and then increased the group numbers until the model best fit the data as identified by the values of BIC and Bayes factors. The results of the model selection are shown in Table 3.

**Figure 2:**
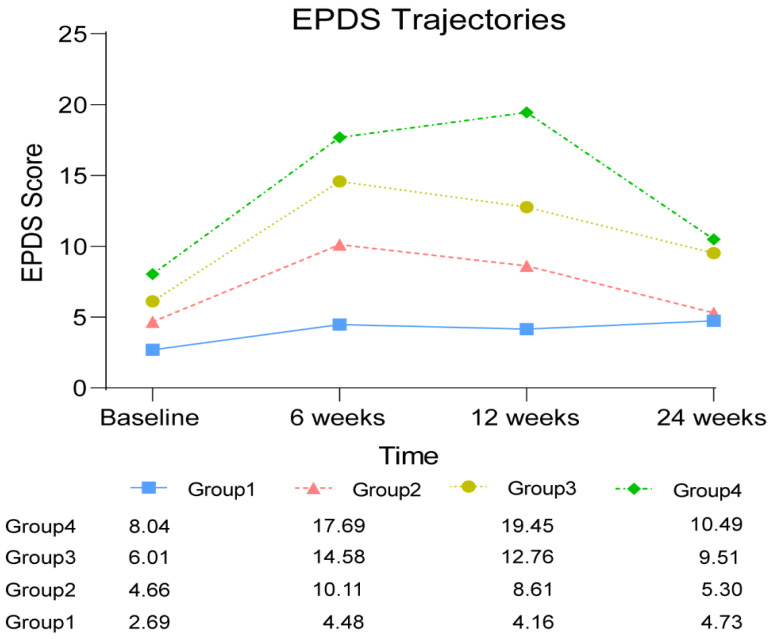
EPDS trajectory.

The BIC value for the model with four trajectory groups was the highest (−4130.93), and the Bayes factor was >1000 when compared to the three-group model. Therefore, the model with four groups was identified, and the model was then refined until the highest cubic polynomial coefficient for each trajectory group was significantly different from zero (*p* < 0.05). After identifying the best model, each subject was assigned to the group with the highest posterior probability of membership. The best model with the four-trajectory group and cubic order is described in Figure 2.

The best model was found in the research to involve four groups that shared similar EPDS trajectories. The trajectory of Group 1 comprised 76 (18.0%) women and was labeled as having “few or no symptoms”, with EPDS scores of less than 5 during a six-month postpartum period. The second group, approximately 31.6% of the population, was labeled as “subclinical but present symptoms”. In the trajectory of Group 2, the 139 new mothers with mean EPDS scores of 4.66–10.01 peaked in the range of mild PPD but mostly stayed in the minimal range at the four different time points. Only at six weeks postpartum did the EPDS score indicate that women in Group 2 had minor PPD. The third group was estimated to include 138 (31.2%) women labeled as having “minor PPD status”. The EPDS scores in the trajectory of Group 3 started with an average of 6.08 at baseline, increased to a peak of 14.66 at six weeks postpartum, and then decreased to 12.83 at 12 weeks postpartum. Finally, there was a group labeled “major PPD status”, comprising 19.2% (*n* = 82) of the population, who displayed high levels of PPD throughout the observation period of six months postpartum. The mean EPDS scores of the Group 4 trajectory dramatically increased to 17.73 at six weeks postpartum from the baseline score of 8.09 and continued to reach a peak of 19.59 at 12 weeks postpartum and then decreased to 10.51 at 24 weeks postpartum. 

The evaluation of model adequacy is described in Table 4. For all four trajectory groups, the lowest AvePP was 0.91, much higher than the recommended value of 0.70. The lower OCC was 20.83, far greater than the recommendation of 5. The values of π_j_ and P_j_ for all groups were similar. The above indices consistently indicate that the model fits the data well.

### 3.3. Predictors of PPD Trajectory Membership

Minor and major PPD trajectory memberships (Group 3 and Group 4) were used as categorical variables in a multinomial logistic regression model to identify predictors of minor and major PPD trajectory membership. The factors associated with PPD status trajectories are described in Table 5. The findings suggested that less degree of support received after childbirth and not attending the parenting training were associated with membership in the minor and major PPD status trajectories compared with the better mental health status trajectory.

## 4. Discussion

To the best of our knowledge, this is the first study to identify the subgroups of Chinese primiparous women that share similar patterns of PPD and to explore the predictors of PPD trajectory membership for Chinese primiparous women. The research findings highlight the advantages of group-based analysis for longitudinal developmental trajectory modeling and evaluate the causes/consequences of the trajectories [23,33]. Using this statistical method, the researchers initiatively identified four groups of Chinese primiparous women that share similar patterns of postpartum depression symptoms that were unknown in the existing literature.

The research found that 18.0% of participants belonged to Group 1, labeled as having “few or no symptoms”, and the first trajectory almost remained stable, with a mean EPDS score of less than 5 during the six-month postpartum period. Around one-third of the subjects fell into the second trajectory labeled as “subclinical but present symptoms”, and the women in Group 2 peaked in the range of mild PPD but mostly stayed in the minimal range at the four different time points. Group 3 included 31.2% of women, labeled as having “minor PPD status”, and the mean score of EPDS in the third trajectory increased to the peak of 14.66 at six weeks postpartum and then decreased to almost 10.00 over the rest of the follow-up period. Finally, there was a group labeled “major PPD status”, comprising 19.2% of the population, who displayed the highest levels of EPDS throughout the observation period of six months postpartum. The EPDS scores of the Group 4 trajectory dramatically increased to 17.73 at six weeks postpartum and continued to reach a peak of 19.59 at 12 weeks postpartum, and then decreased to 10.51 at 24 weeks postpartum. These results indicated that a high percentage of subjects belonged to the minor and major PPD status trajectories, as approximately half of the primiparous women fell into Group 3 and Group 4. The large number of new mothers in the two groups with minor and major PPD status should be given more attention and awareness from health professionals and researchers. Understanding predictors of group membership could help health providers to identify folks to prioritize getting them connected to care. Moreover, timely interventions tailored to these primiparous women belonging to the minor and major PPD trajectories are strongly recommended in further research. Especially women who are likely to be in the major PPD trajectories can be identified early and provided more support.

In terms of developmental trends, the six weeks postpartum period witnessed the peak in EPDS in the three groups. For instance, the trajectories of Groups 1, 2, and 3 were characterized by an initial increase in EPDS score from the baseline to six weeks postpartum, followed by a decrease over the follow-up period. The results from the three groups were aligned with previous studies [14], in which PPD had been reported to have a peak incidence at around six to eight weeks postpartum [38,39]. It is interesting to note that Group 4, with the major PPD status trajectory, displayed a new developmental trend of PPD, inconsistent with prior findings. The results from Group 4 showed that subjects with major PPD status experienced a longer-term EPDS increase and reached a peak at 12 weeks postpartum. Health agencies in the USA [40] and the UK [41] have recommended that regular screening of postpartum women should be conducted to detect PPD symptoms. Recently, the National Health Commission of China (NHC) issued a work plan to introduce screening for depression during pregnancy and childbirth with routine pregnancy tests and postpartum visits during the period of seven weeks (42nd day) postpartum [42]. However, the research findings suggested that the regular, routine screening of PPD should be conducted in the longer term, i.e., at least 12 weeks postpartum, especially for new mothers in the major PPD status trajectory. Interestingly, Tully et al. mentioned the concept of “the fourth trimester” as they found that many American new mothers were not scheduled for follow-up care for 6 weeks after childbirth and had many unmet maternal health needs during the critical transition period [43]. Therefore, the Fourth Trimester Project in the USA was conducted to explore what families need most from birth to 12 weeks postpartum to improve maternal care [43]. Our findings reinforce previous work. Specifically, a study conducted in an American context also identified the first 12 weeks, termed “the fourth trimester” at the time, as a particular need for postpartum individuals.

Findings suggest that level of social support and history of attending parent training were predictors of postpartum depression symptoms at both the major and minor levels. To be more specific, if primiparous women received more degrees of all kinds of support, they were less likely to be on a more severe trajectory, and if primiparous women did not attend parenting training, they were more likely to be on a more severe trajectory. Such predictors may be useful as targets for interventions tailored to primiparous women belonging to the clinically worst PPD trajectories [18].

Social support was identified to positively influence the mental health status of postpartum women, well aligned with previous studies [13,14]. For example, an increased degree of social support of all kinds was found to be related to a decreased risk of deterioration from a normal state to severe PPD, and an increased degree of informational support and evaluation of support were associated with alleviation from severe PPD to a normal state [44]. Furthermore, social support was identified as a predictor of trajectory group membership in the research, which could be related to the Chinese cultural context of “doing the month”. “Doing the month” is derived from Chinese traditional medicine beliefs and has been practiced by Chinese women for more than 1000 years. During the period of “doing the month”, women were asked to eat good nutritious food, avoid wind or cold water, limit their activity at home, avoid any physical work, and were usually accompanied by their mother-in-law or their mother for support [45]. Our previous study found that “doing the month” was still popular in modern society as most Chinese women thought that “doing the month” after childbirth was necessary and still followed these traditional practices [44]. Even though “doing the month” has led to much debate, Chinese women did acquire more support and help from their family members after childbirth than most women in Western countries [45]. It needs to be noted that Chinese primiparous women were reported to lack appropriate informational and appraisal support from health professionals, especially in comparison with the emotional support and material support received from their family members [13,14]. Therefore, in order to improve the mental health status of new mothers, health professionals should play a more active and important role in PPD prevention and alleviation via offering professional informational and appraisal support.

Additionally, attendance at parenting training at the hospital was the most important factor associated with the minor and major PPD status trajectories. This indicates that hospital parenting training had a positive effect on postnatal women’s mental health outcomes. However, only half of the women in the study attended the training, owing to the limited time available among new mothers and the COVID-19 pandemic restrictions throughout society. Thus, more accessible parenting training using various effective methods, e.g., online to offline, is recommended to attract more new mothers to attend hospital training under normalized COVID-19 pandemic prevention and control measures in China. 

This study could be considered within the context of several limitations. Firstly the study was conducted in a Chinese cultural context which prescribes a specific and more hands-on approach to supporting postpartum individuals. Thus findings related to the impact of social support may vary in other cultural contexts. Secondly, owing to financial and time limitations, the study observed the PPD trend up to 24 weeks postpartum. A longer follow-up period may have provided more statistical power to explore more dynamic trajectories. Thirdly, this study only focused on women with normal mental status at baseline and excluded individuals with a depression and anxiety history. Fourthly, as the GBTM remains relatively new in health research, few guidelines exist to help users in reporting model results for trajectory analysis [23,46]. This clearly warrants further research to ensure that adequate information is presented for the critical appraisal of results and comparison between studies. 

## 5. Conclusions

Based on the group-based trajectory model (GBTM), the research identified, for the first time, four groups of Chinese primiparous women that share similar patterns of postpartum depression symptoms and the predictors of minor and major PPD trajectory membership, which were unknown in the existing literature. Almost half of the new mothers were found to fall into the two groups with minor and major PPD status trajectories, and this high proportion of women should be given greater attention and awareness among health professionals and researchers. Understanding predictors of group membership could help health providers to identify folks to prioritize getting them connected to care. Less degree of received support of all kinds, including emotional support, material support, informational support, and evaluation support, and not attending parenting training at the hospitals were identified to predict minor and major trajectory membership. Such predictors can serve as an important reference for health professionals to implement targeted interventions tailored to primiparous women belonging to the minor or major PPD trajectories, which has profound clinical and practical significance.

Moreover, our research provides an indication that the regular, routine screening of PPD should be conducted in the longer term, i.e., at least 12 weeks postpartum, not merely during the period of pregnancy and seven weeks postpartum, as in current clinical practice, especially for new mothers in the major PPD status trajectory. From the findings of this study, there is an identified need for alterations to be made to the current parenting training offered in hospital settings to postnatal women. More accessible parenting training using various effective methods, e.g., online to offline, is recommended to attract more new mothers to attend the programs under the normalized COVID-19 pandemic prevention and control measures in China.

## Figures and Tables

**Table 1:** Missing data distribution.

Missing Data Points	Samples Available for Analysis (%)
1	131 (30.1)
0	304 (69.9)
Total	435 (100.00)

Data points: 3 days, 6 weeks, 12 weeks, and 24 weeks postpartum.

**Table 2:** The social–demographic and clinical characteristics of participants (*n* = 435).

Variables	Total (*n* = 435)
Childbirth age, mean (SD)	25.56 (3.34)
Marriage, *n* (%)	
Married	435 (100%)
Education, *n* (%)	
Middle school or lower	109 (25.06%)
High school	123 (28.28%)
University or higher	203 (46.67%)
Occupation, *n* (%)	
Professional	19 (4.37%)
Skilled	32 (7.36%)
Unskilled	260 (59.78%)
Unemployed	124 (28.51%)
Family income (per month, person) ※, *n* (%)	
<3000 yuan (USD 420)	92 (21.15%)
3001–5000 yuan (USD 420–700)	202 (46.44%)
>5000 yuan (USD 700)	141 (32.41%)
Childbirth mode, *n* (%)	
Natural childbirth	285 (65.52%)
Assisted childbirth *	67 (15.40%)
C-section	83 (19.08%)
Attending parenting training, *n* (%)	
Yes	230 (52.87%)
No	205 (47.13%)
Baby gender, *n* (%)	
Girl	176 (40.46%)
Boy	259 (59.54%)
Baby health, mean (SD)	81.94 (15.09)
Baby fussiness, mean (SD)	66.19 (21.54)
Emotional support, mean (SD)	10.34 (2.74)
Material support, mean (SD)	10.12 (3.28)
Informational support, mean (SD)	6.94 (3.21)
Evaluation of support, mean (SD)	8.55 (2.99)

**※** The average family income (per month, person) was about 4000 yuan (USD 558); * Assisted childbirth was defined as the use of special instruments such as forceps and ventouse to deliver a baby vaginally. The various types of social support were measured by the PSSS, with a range of 0–15 score.

**Table 3:** Model selection results based on all available data points.

Number of Groups	Polynomial Order	BIC	Bayes Factor
1	3	−4658.88	
2	3 3	−4299.13	>1000
3	3 3 3	−4192.20	>1000
4	3 3 3 3	−4130.93	>1000
5	3 3 2 3 2	−4136.80	0.0028

BIC: Bayesian information criterion.

**Table 4:** Model adequacy results.

	Group 1 (*n* = 76)	Group 2 (*n* = 139)	Group 3 (*n* = 138)	Group 4 (*n* = 82)
AvePP	0.94	0.91	0.91	0.96
$P_{j}$	0.18	0.32	0.32	0.19
$\pi_{j}$	0.18	0.32	0.31	0.19
$|\pi_{j}-P_{j}$|	0.01	0.00	0.01	0.00
$OCC_{j}$	69.02	20.83	23.45	93.66

**Table 5:** Multinomial logistic regression predicting factors associated with PPD status trajectory membership.

	Major PPD Status Group (Group 4)			Minor PPD Status Group (Group 3)		
Variables	OR	95% CI	*p* Value	OR	95% CI	*p* Value
Emotional support	0.63	(0.55, 0.73)	<0.001	0.57	(0.50, 0.65)	<0.001
Material support	0.77	(0.69, 0.86)	<0.001	0.75	(0.68, 0.83)	<0.001
Informational support	0.73	(0.66, 0.82)	<0.001	0.69	(0.62, 0.76)	<0.001
Evaluation of support	0.48	(0.41, 0.56)	<0.001	0.61	(0.54, 0.69)	<0.001
Attending parenting training	0.08	(0.04, 0.18)	<0.001	0.14	(0.07, 0.26)	<0.001

Group 1 is the reference group. The minor PPD status group (Group 3) and major PPD status group (Group 4) are compared to Group 1. CI: confidence interval.

## Data Availability

The data presented in this study are available on request from the corresponding author. The data are not publicly available due to privacy restrictions.

## References

[1] Cox J.L., Murray D., Chapman G. (1993). A Controlled Study of the Onset, Duration and Prevalence of Postnatal Depression. Br. J. Psychiatry.

[2] Craig M., Howard L. (2009). Postnatal depression. BMJ Clin. Evid..

[3] El-Gilany A.-H., Elkhawaga G.O., Sarraf B.B. (2018). Depression and its associated factors among elderly: A community-based study in Egypt. Arch. Gerontol. Geriatr..

[4] Radzi C.W.J.B.W.M., Jenatabadi H.S., Samsudin N. (2021). Postpartum depression symptoms in survey-based research: A structural equation analysis. BMC Public Health.

[5] Zeng N., Pope Z., Lee J., Gao Z. (2018). Virtual Reality Exercise for Anxiety and Depression: A Preliminary Review of Current Research in an Emerging Field. J. Clin. Med..

[6] Daley A., Jolly K., MacArthur C. (2009). The effectiveness of exercise in the management of post-natal depression: Systematic review and meta-analysis. Fam. Pract..

[7] Goweda R., Metwally T. (2020). Prevalence and associated risk factors of postpartum depression: A cross sectional study. Arch. Clin. Psychiatry.

[8] Zejnullahu V.A., Ukella-Lleshi D., Zejnullahu V.A., Miftari E., Govori V. (2021). Prevalence of postpartum depression at the clinic for obstetrics and gynecology in Kosovo teaching hospital: Demographic, obstetric and psychosocial risk factors. Eur. J. Obstet. Gynecol. Reprod. Biol..

[9] Zheng X., Morrell J., Watts K. (2018). Changes in maternal self-efficacy, postnatal depression symptoms and social support among Chinese primiparous women during the initial postpartum period: A longitudinal study. Midwifery.

[10] Dadi A.F., Miller E.R., Mwanri L. (2020). Postnatal depression and its association with adverse infant health outcomes in low- and middle-income countries: A systematic review and meta-analysis. BMC Pregnancy Childbirth.

[11] Slomian J., Honvo G., Emonts P., Reginster J.-Y., Bruyère O. (2019). Consequences of maternal postpartum depression: A systematic review of maternal and infant outcomes. Women’s Health.

[12] Puente C.P., Suso-Ribera C., Rico S.B., Marín D., Montero J.S.R., Catalá P. (2021). Is the Association between Postpartum Depression and Early Maternal–Infant Relationships Contextually Determined by Avoidant Coping in the Mother?. Int. J. Environ. Res. Public Health.

[13] Wang Q., Zhang Y., Li X., Ye Z., Huang L., Zhang Y., Zheng X. (2021). Exploring Maternal Self-Efficacy of First-Time Mothers among Rural-to-Urban Floating Women: A Quantitative Longitudinal Study in China. Int. J. Environ. Res. Public Health.

[14] Zheng X., Morrell J., Watts K. (2017). A quantitative longitudinal study to explore factors which influence maternal self-efficacy among Chinese primiparous women during the initial postpartum period. Midwifery.

[15] Woldeyohannes D., Tekalegn Y., Sahiledengle B., Ermias D., Ejajo T., Mwanri L. (2021). Effect of postpartum depression on exclusive breast-feeding practices in sub-Saharan Africa countries: A systematic review and meta-analysis. BMC Pregnancy Childbirth.

[16] Wilkinson R.B., Mulcahy R. (2010). Attachment and interpersonal relationships in postnatal depression. J. Reprod. Infant Psychol..

[17] Nagin D.S. (2014). Group-Based Trajectory Modeling: An Overview. Ann. Nutr. Metab..

[18] Nguefack H.L.N., Pagé M.G., Katz J., Choinière M., Vanasse A., Dorais M., Samb O.M., Lacasse A. (2020). Trajectory Modelling Techniques Useful to Epidemiological Research: A Comparative Narrative Review of Approaches. Clin. Epidemiol..

[19] Baron E., Bass J., Murray S.M., Schneider M., Lund C. (2017). A systematic review of growth curve mixture modelling literature investigating trajectories of perinatal depressive symptoms and associated risk factors. J. Affect. Disord..

[20] Barban N., Billari F.C. (2012). Classifying life course trajectories: A comparison of latent class and sequence analysis. J. R. Stat. Soc. Ser. C (Applied Stat.).

[21] Frankfurt S., Frazier P., Syed M., Jung K.R. (2016). Using Group-Based Trajectory and Growth Mixture Modeling to Identify Classes of Change Trajectories. Couns. Psychol..

[22] Nagin D.S., Odgers C.L. (2010). Group-Based Trajectory Modeling in Clinical Research. Annu. Rev. Clin. Psychol..

[23] Song M. (2019). Trajectory analysis in obesity epidemiology: A promising life course approach. Curr. Opin. Endocr. Metab. Res..

[24] Cox J.L., Holden J.M., Sagovsky R. (1987). Detection of Postnatal Depression: Development of the 10-item Edinburgh Postnatal Depression Scale. Br. J. Psychiatry.

[25] Cox J., Holden J. (2003). Perinatal Mental Health: A Guide to the Edinburgh Postnatal Depression Scale (EPDS).

[26] Levis B., Negeri Z., Sun Y., Benedetti A., Thombs B.D. (2020). Accuracy of the Edinburgh Postnatal Depression Scale (EPDS) for screening to detect major depression among pregnant and postpartum women: Systematic review and meta-analysis of individual participant data. BMJ.

[27] O’Connor E., Rossom R.C., Henninger M., Groom H.C., Burda B.U. (2016). Primary Care Screening for and Treatment of Depression in Pregnant and Postpartum Women: Evidence Report and Systematic Review for the US Preventive Services Task Force. JAMA.

[28] Wang Y., Guo X., Lau Y., Chan K.S., Yin L., Chen J. (2009). Psychometric evaluation of the Mainland Chinese version of the Edinburgh Postnatal Depression Scale. Int. J. Nurs. Stud..

[29] Lu H., Zheng X. (2001). The relationship between social support and postnatal depression of primiparous women. Chin. J. Nurs..

[30] Arrandale V., Koehoorn M., MacNab Y., Kennedy S.M. (2006). How to Use SAS^®^ Proc Traj and SAS^®^ Proc Glimmix in Respiratory Epidemiology.

[31] Jones B.L., Nagin D.S. Proc TRAJ: A SAS Procedure for Group-Based Modeling of Longitudinal Data. Proceedings of the 135st APHA Annual Meeting and Exposition 2007.

[32] Twisk J., Hoekstra T. (2012). Classifying developmental trajectories over time should be done with great caution: A comparison between methods. J. Clin. Epidemiol..

[33] Niyonkuru C., Wagner A.K., Ozawa H., Amin K., Goyal A., Fabio A. (2013). Group-Based Trajectory Analysis Applications for Prognostic Biomarker Model Development in Severe TBI: A Practical Example. J. Neurotrauma.

[34] Jamshidian M., Jalal S. (2010). Tests of Homoscedasticity, Normality, and Missing Completely at Random for Incomplete Multivariate Data. Psychometrika.

[35] Nagin D. (2005). Group-Based Modeling of Development.

[36] Wagner A.K., Amin K.B., Niyonkuru C., A Postal B., McCullough E.H., Ozawa H., Dixon C.E., Bayir H., Clark R.S., Kochanek P. (2011). CSF Bcl-2 and cytochrome C temporal profiles in outcome prediction for adults with severe TBI. J. Cereb. Blood Flow Metab..

[37] Wagner A.K., McCullough E.H., Niyonkuru C., Ozawa H., Loucks T., Dobos J.A., Brett C.A., Santarsieri M., Dixon C.E., Berga S.L. (2011). Acute Serum Hormone Levels: Characterization and Prognosis after Severe Traumatic Brain Injury. J. Neurotrauma.

[38] Gavin N.I., Gaynes B.N., Lohr K.N., Meltzer-Brody S., Gartlehner G., Swinson T. (2005). Perinatal depression: A systematic review of prevalence and incidence. Obstet. Gynecol..

[39] Hewitt C., Gilbody S., Brealey S., Paulden M., Palmer S., Mann R., Green J., Morrell J., Barkham M., Light K. (2009). Methods to identify postnatal depression in primary care: An integrated evidence synthesis and value of information analysis. Health Technol. Assess..

[40] Siu A.L., Bibbins-Domingo K., Grossman D.C., Baumann L.C., Davidson K., Ebell M., García F.A.R., Gillman M., Herzstein J., Kemper A.R. (2016). Screening for Depression in Adults: US Preventive Services Task Force Recommendation Statement. JAMA.

[41] Health NCCfM (2014). Antenatal and Postnatal Mental Health: Clinical Management and Service Guidance.

[42] Gong W., Jin X., Cheng K.K., Caine E.D., Lehman R., Xu D.R. (2020). Chinese Women’s Acceptance and Uptake of Referral after Screening for Perinatal Depression. Int. J. Environ. Res. Public Health.

[43] Tully K.P., Stuebe A.M., Verbiest S.B. (2017). The fourth trimester: A critical transition period with unmet maternal health needs. Am. J. Obstet. Gynecol..

[44] Xiong J., Fang Q., Chen J., Li Y., Li H., Li W., Zheng X. (2021). States Transitions Inference of Postpartum Depression Based on Multi-State Markov Model. Int. J. Environ. Res. Public Health.

[45] Zheng X., Watts K., Morrell J. (2018). Chinese primiparous women’s experience of the traditional postnatal practice of “Doing the month”: A descriptive method study. Jpn. J. Nurs. Sci..

[46] van de Schoot R., Sijbrandij M., Winter S.D., Depaoli S., Vermunt J.K. (2016). The GRoLTS-Checklist: Guidelines for Reporting on Latent Trajectory Studies. Struct. Equ. Model. A Multidiscip. J..

